# Metal ions in macrophage antimicrobial pathways: emerging roles for zinc and copper

**DOI:** 10.1042/BSR20130014

**Published:** 2013-07-16

**Authors:** Sian L. Stafford, Nilesh J. Bokil, Maud E. S. Achard, Ronan Kapetanovic, Mark A. Schembri, Alastair G. McEwan, Matthew J. Sweet

**Affiliations:** *School of Chemistry and Molecular Biosciences, The University of Queensland, Brisbane, Queensland 4072, Australia; †The Australian Infectious Diseases Research Centre, The University of Queensland, Brisbane, Queensland 4072, Australia; ‡Institute for Molecular Bioscience, The University of Queensland, Brisbane, Queensland 4072, Australia

**Keywords:** copper transporter (CTR), host defence, innate immunity, monocyte, Toll-like receptor, zinc transporter, CP, ceruloplasmin, CTR, copper transporter, DC, dendritic cells, FPN1, ferroportin 1, GPI, glycosylphosphatidylinositol, IFNγ, interferon γ, IKKβ, IκB (inhibitor of nuclear factor κB) kinase β, IL, interleukin, LPS, lipopolysaccharide, MAPK, mitogen-activated protein kinase, MMP, matrix metalloproteinase, MT, metallothionein, NF-κB, nuclear factor κB, Nrf2, nuclear factor-erythroid 2-related factor 2, PDE, phosphodiesterase, ROS, reactive oxygen species, SOD, superoxide dismutase, TLR, Toll-like receptor, TNF, tumour necrosis factor, TRIF, TIR (Toll/interleukin-1 receptor) domain-containing adaptor protein inducing interferon β

## Abstract

The immunomodulatory and antimicrobial properties of zinc and copper have long been appreciated. In addition, these metal ions are also essential for microbial growth and survival. This presents opportunities for the host to either harness their antimicrobial properties or limit their availability as defence strategies. Recent studies have shed some light on mechanisms by which copper and zinc regulation contribute to host defence, but there remain many unanswered questions at the cellular and molecular levels. Here we review the roles of these two metal ions in providing protection against infectious diseases *in vivo*, and in regulating innate immune responses. In particular, we focus on studies implicating zinc and copper in macrophage antimicrobial pathways, as well as the specific host genes encoding zinc transporters (SLC30A, SLC39A family members) and CTRs (copper transporters, ATP7 family members) that may contribute to pathogen control by these cells.

## INTRODUCTION

The immune system requires essential micronutrients and trace elements such as iron, zinc, copper and selenium for optimal function [1]. Deficiency in these elements leads to suppression in the activities of cells of the innate and adaptive immune systems, through impaired function and/or decreased cell number. Such defects can lead to increased morbidity and mortality to viral, microbial and parasitic infections, with immunocompetence being restored when the deficiency is reversed [1]. Despite the clear biological relevance of micronutrients to host defence, the cellular and molecular mechanisms by which they regulate host immune function are very much an emerging area of research. Here we review the literature on the beneficial effects of zinc and copper in host defence, as well as recent evidence linking copper and zinc directly to macrophage antimicrobial pathways. Certain parallels suggest that there may be common mechanisms by which they contribute to these responses. We do not extensively cover strategies utilized by pathogens to defend against zinc- and copper-mediated host defence, since others have reviewed this literature in detail recently [2–4]. Similarly, we provide only a brief overview of the effects of zinc on signalling and gene expression in monocytes and macrophages; while clearly important for host defence, this area has been covered by other excellent reviews [5–7]. Rather, the major objective of this review is to summarize emerging evidence for direct contributions of zinc and copper to macrophage antimicrobial responses, to highlight the likely significance of this to host defence, and to identify the major knowledge gaps in this area, particularly relating to the roles of specific host zinc and copper transport genes.

### Zinc deficiency compromises immune function and host defence

Zinc is essential for the growth and development of most organisms [8], and in humans, it is the second most abundant transition metal ion [9]. Studies of zinc deficiency in humans, as well as animal models, have conclusively demonstrated that zinc is essential for normal immune function, but its precise cellular and molecular role(s) remain enigmatic. Zinc deficiency was first identified as a clinical syndrome in the Middle East approximately 50 years ago [10]. The first case was reported in Iran in a male patient who presented with severe anaemia and growth retardation [11]. This condition was associated with hepatosplenomegaly, hypogonadism, rough and dry skin, mental lethargy and genophagia (clay eating), and was initially misdiagnosed as iron-deficiency anaemia. Similar cases were reported in Egypt [12], where a regimen of zinc supplementation reversed hypogonadism and growth retardation [13]. In addition to developmental anomalies and anaemia, the patients also suffered from severe immune deficiency leading to intercurrent infection and death before the age of 25 [8]. Furthermore, experimentally induced mild zinc deficiency in human subjects [14] resulted in a decrease in the ratio of CD4^+^ to CD8^+^ T-cells, and a decreased Th1 response as assessed by IFNγ (interferon γ), IL (interleukin)-2 and TNFα (tumour necrosis factor α) secretion [15]. It was proposed that defective Th1 responses compromised host defence to intracellular parasitic infections, in particular. In general, severe zinc deficiency is rare, but mild-to-moderate deficiency is quite common throughout the world especially in developing countries, lower socio-economic groups and in individuals with chronic alcoholism. The 2002 WHO world health report attributed about 800 000 deaths (1.4% of global mortalities) to zinc deficiency, and estimated that it contributed to 16% of lower respiratory tract infections, 18% of malaria and 10% of diarrhoeal disease [16]. Animal models of zinc deficiency have also been used to assess the role of zinc in regulating the immune system and host defence. Early studies examined the effect of zinc deficiency on T-cell function in young A/J mice [17,18]. Thymic atrophy and diminished antigen-specific T-cell responses were observed, which was reversed by dietary zinc supplementation. In a mouse polymicrobial model of sepsis, zinc deficiency resulted in increased bacterial burdens in the blood, a heightened pro-inflammatory response, and increased mortality, and these effects were reversed by zinc supplementation [19,20]. The enhanced inflammatory response correlated with increased activity of NF-κB (nuclear factor κB) and expression of NF-κB-dependent target genes [19], suggesting that zinc acts to constrain this pathway during excessive inflammatory responses.

Zinc deficiency generally reflects insufficient dietary intake, but systemic inflammation also results in a reduction in the levels of circulating zinc (hypozincaemia). Indeed, it has long been appreciated that plasma zinc levels decline during systemic bacterial infections [21], as a physiological component of the acute phase response [22]. Hypozincaemia as a consequence of LPS (lipopolysaccharide) challenge has been experimentally demonstrated in humans. LPS administration to healthy human volunteers reduced serum zinc levels, without increasing zinc loss in the urine or decreasing levels of the major zinc-binding protein, serum albumin [23]. This suggests that the drop in circulating zinc reflects its redistribution elsewhere. Evidence from animal models suggests that increased uptake of zinc by hepatocytes in the liver is the mechanism responsible. Yasuno et al. [24] showed that when rats were given an intravenous dose of zinc (50 *μ*g/kg), the metal was rapidly distributed to the liver, spleen, intestine, kidney and pancreas. Moreover, systemic inflammation in mice resulted in IL-6-dependent up-regulation of the zinc importer *Slc39a14*, which enabled zinc uptake by hepatocytes in the liver [25,26]. Several studies have documented reduced serum zinc levels in various patient cohorts. For example, zinc levels in polymorphonuclear cells from hospitalized elderly patients were reduced by comparison with those of healthy elderly subjects [27]. A more recent study demonstrated that plasma zinc concentrations were decreased in critically ill patients, and were dramatically lower in patients with septic shock [28]. This suggests that hypozincaemia during systemic inflammation in humans may contribute to pathology. Thus it appears that, in both human and animal models, dietary zinc deficiency predisposes to infectious diseases, while hypozincaemia may also contribute to morbidity and mortality during severe infections.

### Benefits of zinc supplementation in host defence

Zinc supplementation has demonstrated beneficial effects in infectious disease, both clinically and in animal models. More than three decades ago, Snyder and Walker [29] showed that ZnCl_2_ administration to mice 1 h before LPS challenge offered almost complete protection against an otherwise lethal dose. Improved survival and reduced bacterial loads were also observed in a polymicrobial sepsis model when C57Bl/6 mice received prophylactic zinc gluconate treatment [30]. Similarly, zinc supplementation decreased the parasite load in the blood in Wistar rats infected with *Trypanosoma cruzi* [31], and restored the ability of alcohol-fed animals to clear *Klebsiella pneumoniae* from the lung [32]. Beneficial effects of zinc administration in promoting pathogen clearance and/or reducing pathology have also been reported in a mouse *Candida albicans* infection model [33], a rat model of *Escherichia coli*-mediated prostatitis [34], and a Rhesus monkey model of severe diarrhoeal disease caused by enteropathogenic *E. coli* [35]. More importantly, many studies have demonstrated that zinc supplementation has beneficial effects in clinical settings, particularly in severe diarrhoeal diseases and respiratory tract infections. Pooled analysis of randomized controlled trials of zinc supplementation in children suffering from severe diarrhoea and pneumonia showed that this treatment regime reduced the incidence of pneumonia by 41% and the incidence of diarrhoea by 18% and its prevalence by 25% [36]. Similarly, a meta-analysis of 22 independent studies demonstrated that oral zinc supplementation reduced the frequency and duration of acute and persistent diarrhoea in infants by up to 18% [37]. Indeed, a WHO and UNICEF report recommended the inclusion of zinc in oral rehydration solution to treat gastroenteritis in infants and children [38]. Randomized placebo control trials on children with severe pneumonia also showed that zinc supplementation (2 mg/kg per day) reduced the length of hospital stay and the severity of infection [39]. Zinc supplementation has also been reported to show beneficial effects for a range of other infectious diseases including shigellosis, leprosy, tuberculosis and leishmaniasis [40]. Finally, Mocchegiani et al. [41] reported that zinc supplementation reduced opportunistic infections in HIV-infected patients, although the beneficial effect was restricted to certain pathogens (*Pneumocystis carinii* and *C. albicans*) and not others [CMV (cytomegalovirus), *Toxoplasma*]. Indeed, zinc supplementation for the treatment of infectious diseases was not efficacious in all infectious disease trials. For example, zinc supplementation in a randomized placebo control trial among Polish children suffering from acute gastroenteritis [42], and zinc and vitamin A supplementation in pulmonary tuberculosis showed no therapeutic benefit [43]. Thus, zinc supplementation may be effective in controlling specific infections only in individuals with notable zinc deficiency, or alternatively, other environmental and/or genetic factors may impact on efficacy of this treatment. Furthermore, zinc supplementation may actually exacerbate disease severity for some pathogens. Vitamin A and zinc supplementation was assessed in gastrointestinal parasitic infections among Mexican children [44]. Zinc reduced the incidence of *Giardia lamblia* and *Entamoeba histolytica* infections, but increased *Ascaris lumbricoides*-associated diarrhoea. Animal model studies also suggest that high-dose zinc supplementation may exert undesirable effects that are independent of immune function; for example, leading to hippocampus-dependent memory impairment [45]. Taken together, the above literature suggests that zinc has an essential role in immune function and protecting against infectious disease, but zinc supplementation is probably only effective in conditions of zinc deficiency, and only for certain infections. Thus, it is essential to understand the exact roles that zinc plays during different infections, and the mechanism(s) by which it acts.

### Zinc and macrophage antimicrobial responses

Zinc exerts a multitude of effects on numerous immune cell types [6]. Nonetheless, many of the studies reporting effects of zinc deficiency or supplementation on infectious disease outcomes also report effects on macrophage numbers or function. This suggests that macrophages may be an important cellular target of zinc action during infections. For example, beneficial effects of zinc supplementation in a mouse model of polymicrobial sepsis were associated with enhanced phagocytosis of *E. coli* and *Staphylococcus aureus* by peritoneal macrophages [30]. Similarly, zinc supplementation increased peritoneal macrophage numbers in a *T. cruzi* infection model, while zinc deficiency impaired the ability of peritoneal macrophages to kill this parasite [31,46]. Several other studies have also reported that zinc promotes macrophage phagocytic capacity and/or pathogen clearance by these cells [47–49]. However, most of these studies have not addressed the molecular mechanisms responsible for such effects. Recent evidence suggests that regulated zinc trafficking within macrophages may play an active role in antimicrobial responses.

The macrophage activating cytokines TNFα and IFNγ promoted the phagosomal accumulation of zinc in *Mycobacterium avium*-infected mouse macrophages, and phagosomal zinc also accumulated over time in response to infection with *Mycobacterium tuberculosis* [50]. Thus, this metal ion can concentrate within the macrophage phagolysosome, where it presumably may contribute to antimicrobial responses. A recent study supported this concept by showing that upon infection of human macrophages, *M. tuberculosis* expressed *ctpC*, which encodes a zinc efflux pump [51]. This would likely be required to cope with a high zinc environment. The same study also reported that *M. tuberculosis* infection triggered the accumulation of free zinc within macrophage phagosomes at 4 h post-infection, and that this zinc co-localized with intracellular bacteria [51]. Such evidence suggests that high levels of zinc may exert direct bactericidal effects within macrophages. The specific mechanisms by which this might occur are unknown, but are most likely to involve essential proteins required for bacterial survival being inactivated, for example by destruction of Fe–S clusters [52]. Competition with other metal ions may also be involved. For example, high concentrations of zinc can starve *Streptococcus pneumoniae* of essential manganese, by competing for binding to the manganese solute binding protein PsaA [53]. Whether similar mechanisms operate for the professional intramacrophage pathogens such as *M. tuberculosis* is unknown. It is also possible that the positive effects of zinc on macrophage responses to pathogen challenge relate to the numerous zinc-containing proteins with roles in host defence. For example, MMPs (matrix metalloproteinases) are zinc-dependent proteases [54], some of which have functions in antimicrobial responses. MMP12, also referred to as macrophage elastase, has direct antimicrobial effects against bacteria within the macrophage phagolysosome. It adheres to bacterial cell walls and disrupts the cell membrane leading to cell death, and this effect was reportedly independent of enzymatic activity [55]. MMP7 is involved in the activation of defensins by cleaving the pro form of α- and β-defensins to the active form [56], which then can have direct antimicrobial effects.

In contrast to the above studies, zinc starvation may also be employed as part of the macrophage response to *Histoplasma capsulatum* [57], a fungal pathogen that can survive intracellularly within these cells. This study showed that zinc chelation restricted *H. capsulatum* growth, and that infection of GM-CSF (granulocyte/macrophage colony stimulating factor)-derived murine peritoneal and bone marrow macrophages with *H. capsulatum* decreased the intracellular zinc concentration. The TLR (Toll-like receptor) 4 agonist LPS from Gram-negative bacteria also reduced the intracellular zinc concentration within mouse DC (dendritic cells) [58], suggesting that zinc export may occur in response to infection by some micro-organisms. Thus, zinc restriction may also be utilized as a macrophage antimicrobial mechanism, somewhat analogous to the antimicrobial effect of zinc chelation by neutrophil-derived calprotectin in the extracellular space [59–61]. Not surprisingly, some pathogens have evolved mechanisms to thwart zinc starvation by the host. For example, *Salmonella enterica* serovar Typhimurium (*S.* Typhimurium) thrives in the inflamed gut by expressing ZnuABC, a high-affinity zinc transporter that overcomes calprotectin-mediated zinc chelation [62]. The utilization of zinc in macrophage antimicrobial pathways versus zinc sequestration as a nutrient starvation strategy to limit microbial growth highlights the complexity of zinc involvement in macrophage functions. Zinc trafficking may have distinct functions in these cells, depending on the specific infectious agent encountered and/or the zinc concentrations that are present. Indeed, the effects of zinc on macrophage inflammatory responses vary in a concentration-dependent manner [5].

### Zinc effects on macrophage signalling and gene expression and links to host defence

**Figure 1:**
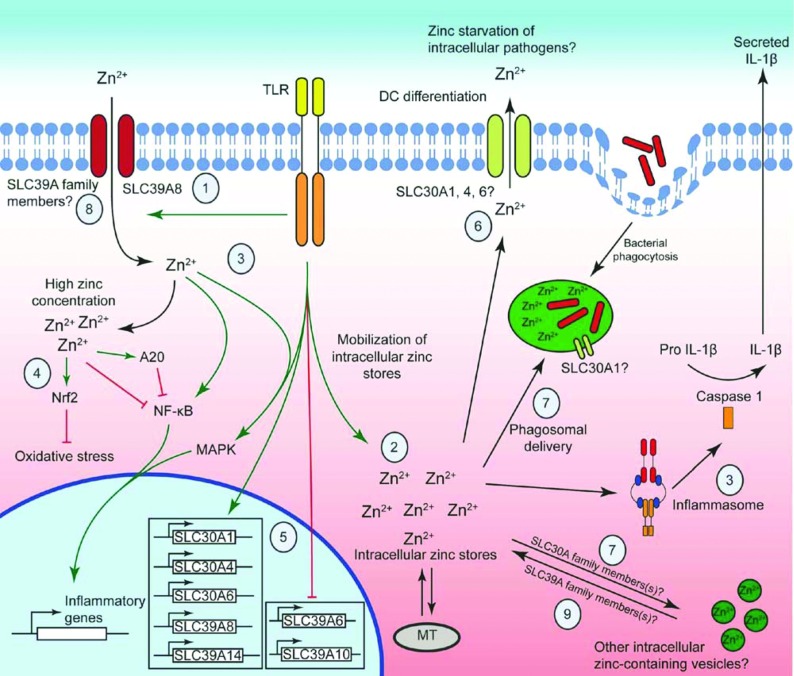
Interplay between zinc trafficking, inflammatory signalling and antimicrobial responses in macrophages TLR4 signalling promotes the rapid accumulation of free zinc (within minutes) within macrophages, an effect that may be mediated by the zinc importer SLC39A8 (1) and/or redistribution of intracellular zinc pools (2). Zinc is required for the activation of many TLR signalling responses and for pannexin-1-dependent inflammasome activation (3). However, high levels of zinc can also inhibit inflammatory signalling pathways in macrophages, for example by inhibiting IKKβ or promoting the expression or activity of Nrf2 and A20 (4). TLR signalling modulates the expression of many zinc transport genes (5) and mobilizes the intracellular zinc pool. TLR-induced SLC30A family members (e.g. SLC30A1, 4 and/or 6) may promote zinc efflux to starve pathogens of zinc (6) and/or deliver zinc to the phagosome and other intracellular vesicles (7) to activate antimicrobial responses (including direct zinc toxicity). Inhibition of expression of some SLC39A family members (e.g. SLC39A6, 10) by TLR signalling may contribute to net zinc export, whereas up-regulation of others (e.g. SLC39A8, 14) may contribute to zinc uptake under some conditions (8) and/or vesicular export of zinc and/or other metal ions to the cytoplasm (9).

While the above emerging literature implicates regulated zinc trafficking in macrophages in direct antimicrobial responses and/or nutrient starvation, a much more substantial literature has documented effects of zinc on signalling and inflammatory outputs in immune cells, including monocytes and macrophages [5,6]. Indeed, approximately 5% of genes were reported to be zinc-responsive in a human monocytic cell line [63]. Such effects are likely to be an important component of zinc-mediated host defence. Zinc regulates inflammatory gene expression through multiple pathways including protein tyrosine phosphorylation, MAPKs (mitogen-activated protein kinases), PKC (protein kinase C), PDEs (phosphodiesterases) and NF-κB [5,6], many of which lie downstream of TLRs. Indeed, much of the focus relating to zinc signalling in monocytes and macrophages has been on TLR signalling. Relatively few studies have assessed other pathways such as Nod-like receptor-mediated activation of the inflammasome, a large intracellular signalling platform that processes caspase-1 leading to maturation of caspase-1-dependent cytokines such as IL-1β [64]. However, one study has reported that IL-1β release downstream of the NLRP3 inflammasome was dependent on zinc [65]. Hence, zinc may contribute to macrophage-mediated host defence through promoting inflammasome activation, in addition to regulating TLR responses. A reoccurring theme in zinc signalling is that zinc concentrations are critical; while low concentrations may be required for the activation of a specific pro-inflammatory signalling pathway, high concentrations can suppress the same pathway [5]. How regulated zinc trafficking in macrophages (introduced above) intersects with effects on inflammatory signalling and gene expression in these cells requires more detailed investigation, but existing evidence suggests that distinct temporal changes may be important. LPS triggers a rapid and transient accumulation of free zinc in human and mouse monocytes/macrophages (within minutes), and this effect was required for the activation of pro-inflammatory signalling pathways in these cells [66]. However, LPS decreased the intracellular zinc levels in DC in a TRIF [TIR (Toll/interleukin-1 receptor) domain-containing adaptor protein inducing interferon β]-dependent manner over a longer time course (several hours), and this was required for efficient DC maturation, and thus antigen presentation [58]. Thus, time is also likely to be an important factor in dictating intracellular zinc concentrations and how this affects inflammatory responses. The complex interplay between zinc trafficking and macrophage functions is summarized in Figure 1, and below, we briefly outline the existing literature linking zinc to pro- and anti-inflammatory signalling and gene expression in macrophages.

Zinc regulates the pro-inflammatory transcription factor NF-κB. Modest zinc supplementation (15 mg/day) of healthy young men substantially enhanced LPS-induced mRNA expression of the NF-κB-dependent gene TNF in monocytes [67], whereas dietary zinc depletion suppressed LPS-inducible TNF production from whole blood [68]. Given the crucial role of this cytokine in control of infectious diseases, such effects are likely to be important for zinc-mediated host defence. The direct requirement for zinc in activating NF-κB has been demonstrated in T-cells [69], and in response to LPS in human and mouse monocytes/macrophages [66]. However, an extensive literature has also documented inhibitory effects of zinc on NF-κB activation, both *in vitro* [70] and *in vivo* [71]. In the latter case, this also correlated with reduced TNF expression and liver injury, an effect that was MT (metallothionein)-independent. Others have also reported that zinc exerts protective effects *in vivo* by limiting NF-κB activation during innate immune responses [19]. Multiple mechanisms of NF-κB inhibition have been identified. Very recently, direct inhibition of IKKβ [IκB (inhibitor of NF-κB) kinase β] by zinc was reported [72], while others have also shown that zinc indirectly inhibits IKKβ via cGMP-dependent activation of PKA (protein kinase A) [73]. Zinc also up-regulates the expression of *A20*, a negative regulator of inflammatory responses [74]. Divergent effects of zinc have also been noted with respect to PDEs, which require zinc for activity but are inhibited by high concentrations of zinc [5,6]. Various other primary TLR-activated signalling pathways including MAPK p38 and ERK (extracellular-signal-regulated kinase) are also zinc-dependent [66]. Thus, it would seem likely that zinc acts as a key component of many primary TLR signalling events, but also as an important mechanism of feedback control. This basic premise may partly underlie the seemingly opposing pro- and anti-inflammatory effects of zinc on macrophage signalling and gene expression. At least part of the mechanism by which zinc exerts anti-inflammatory effects may also relate to its indirect antioxidant proprieties. Zinc can protect essential thiol-containing proteins by stabilizing them, and can also reduce the formation of ROS (reactive oxygen species) by competition with redox-active transition metals such as Cu^+^ and Fe^2+^ [75]. Zn^2+^ is also an important co-factor of SOD (superoxide dismutase) [76], which plays an important role in the degradation of pro-inflammatory ROS. There are certainly some studies linking protective effects of zinc during inflammation to reduced oxidative stress. Zinc supplementation to alcohol-fed rats restored expression of Nrf2 (nuclear factor-erythroid 2-related factor 2) [32], a transcription factor that protects against oxidative stress and limits host pathology during sepsis [77]. Similarly, the zinc-mediated reduction in LPS-induced liver injury in mice was associated with reduced oxidative stress [71]. Such mechanisms may contribute to the reduced immunopathology associated with zinc supplementation during severe infectious diseases.

### What zinc transporters regulate macrophage functions?

**Figure 2:**
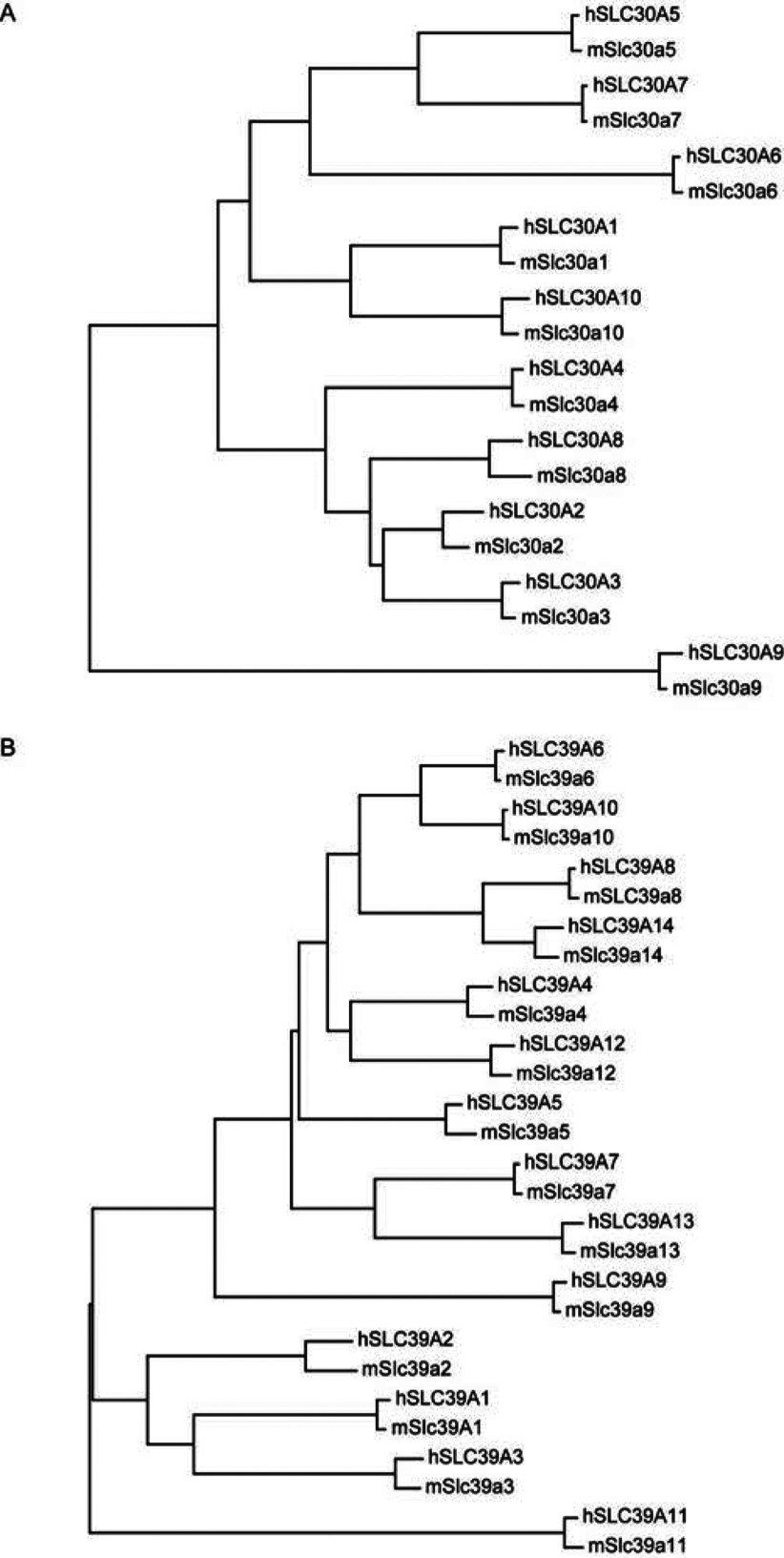
Nearest-neighbour-joining generated phylogenetic tree of human and mouse SLC30A and SLC39A family members Protein sequences for all the family members were obtained from the Ensembl genome browser website (http://www.ensembl.org/) and aligned using clustal omega from EMBL-EBI (http://www.ebi.ac.uk/Tools/msa/clustalo/). The alignment was imported into MEGA5.1 software [152] and a phylogenetic tree was generated.

Zinc homoeostasis is maintained through MT, a family of zinc-binding proteins controlling cellular zinc distribution in a redox-dependent manner [78]. LPS rapidly up-regulates MT expression in human macrophages [79]; presumably this may act to sequester the free intracellular zinc that is generated immediately after TLR activation [66], and would be broadly consistent with the anti-inflammatory roles of MT, as identified by gene knock-out studies [80–82]. Zinc homoeostasis is additionally controlled by two distinct families of zinc transporters, the SLC30A family (ZnT; vertebrate cation diffusion facilitator family) and the SLC39A family [Zip (Zrt/Irt-like proteins)]. SLC30A family members are predicted to have six transmembrane domains, while members of the SLC39A family are predicted to have eight transmembrane domains [83]. Human and mouse orthologues of the ten identified members of the *SLC30A* family and the 14 identified members of the *SLC39A* family are closely related as assessed by phylogenetic analysis of their encoded proteins (Figure 2). A neighbour-joining phylogenetic tree of protein sequences for the SLC30A family shows that human and mouse SLC30A9 diverge from the rest of the family, possibly indicating a disparate function for this family member. Members of the SLC39A family can be divided into three groups with SLC39A11 in one group, SLC39A1, 2 and 3 in another group and the rest of the family (SLC39A4–10, 12 and 13) making up the last group. Within the latter group, SLC39A8 and 14 are closely related, which is interesting in light of studies suggesting roles for both of these genes in innate immune pathways (discussed below). The SLC30A family have generally been linked to zinc efflux from cells, whereas members of the SLC39A family promote zinc influx [83]. Importantly, several SLC39A proteins have been shown to transport other metal ions, so the specificity of individual transporters is likely to be highly dependent on the specific cell-type, as well as the intracellular and extracellular environments. Expression analyses and genetic studies provide some evidence for roles for individual zinc transporters in host defence, but very few functional studies in macrophages are yet to emerge.

#### The SLC30A family

The decrease in intracellular zinc concentrations in innate immune cells in response to *H. capsulatum* [57] and LPS [58] implicate specific SLC30A family members in zinc efflux from macrophages during pathogen challenge. Most of the SLC30A family are expressed in various immune cell populations [84], and it is unknown which specific members are involved in zinc efflux from macrophages. Nevertheless, LPS up-regulated the mRNA expression of *Slc30a1*, *Slc30a4* and *Slc30a6* in murine DC [58], thus presenting these as obvious candidates. Interestingly, this effect was dependent on the TLR adaptor protein TRIF, as was the reduction in intracellular zinc levels. This provides circumstantial evidence that one or more of these transporters may contribute to LPS-triggered zinc efflux from macrophages. *SLC30A1* expression was also up-regulated by *M. tuberculosis* infection in human macrophages [51]. As metal ion exporters, it is possible that one or more of these transporters may also contribute to the accumulation of zinc within macrophage phagosomes in response to *M. tuberculosis* [50,51] and perhaps other infectious agents. Indeed, several members of this family including SLC30A2 [85,86], SLC30A6 [87], SLC30A7 [88] and SLC30A8 have been localized to acidic endosomal/lysosomal vesicles, the *trans*-Golgi network, the Golgi apparatus and intracellular vesicles, respectively, in non-macrophage cell types.

#### The SLC39A family

The SLC39A family are thought to transport metal ions to the cytoplasm from either the extracellular environment or intracellular vesicular compartments. In keeping with the LPS-mediated reduction in intracellular zinc levels in mouse DC, LPS down-regulated mRNA levels of *Slc39a6* and *Slc39a10* in these cells [58]. Furthermore, Slc39a6 overexpression blocked the LPS-triggered reduction in intracellular zinc levels, implying that the down-regulation of these transporters may be required for regulated zinc export in response to Gram-negative bacterial pathogens. Slc39a6 was also recently reported to have functions in T-cells during immunological synapse formation with DC. Knock down of *Slc39a6* expression diminished zinc uptake by activated T-cells, which was required for signalling downstream of the T-cell receptor [89]. Although no studies have yet assessed Slc39a6 function in macrophages, the above reports hint that this transporter may have a role in regulating zinc trafficking, and potentially antimicrobial responses, in these cells.

SLC39A8 has been linked to inflammatory responses in various cell types. It was reported to have a cytoprotective function in respiratory epithelial cells, where it protected these cells from TNFα-induced cytotoxicity [90]. TNFα promoted the translocation of SLC39A8 to the plasma membrane in these cells, probably leading to enhanced zinc uptake and cytoprotection. SLC39A8 function has also been investigated in human T-cells [91]. In these cells, it accumulated within the lysosomal compartment and enhanced zinc-inducible IFNγ production. Importantly, elevated *SLC39A8* mRNA expression in monocytes has also been associated with sepsis severity [28], and more recently SLC39A8 was identified as a gene affecting the course of malaria infection in West African children [92]. These links to infectious disease outcomes and to inflammatory pathways suggest that SLC39A8 may regulate macrophage antimicrobial pathways, although this awaits functional evidence. Given that this transporter can reside at both the cell surface and in lysosomal compartments, it is difficult to predict how it might exactly function in antimicrobial pathways. However, recent work from Liu *et al* did identify a role for this gene in acting as a feedback controller of macrophage inflammatory responses [72]. The authors confirmed that LPS and TNF up-regulated *SLC39A8* expression, and that the mechanism involved direct regulation by the transcription factor NF-κB. The authors also showed that this up-regulation of SLC39A8 enabled enhanced zinc uptake, which acted to directly inhibit IKKβ function. Very recently, evidence for SLC39A14 in macrophage function has emerged. LPS up-regulated *SLC39A14* mRNA in primary human macrophages and this acted to constrain inflammatory responses [93]. *Slc39a14* mRNA and protein was also LPS-inducible in mice, and *Slc39a14*^−/−^ mice have impaired zinc uptake, altered plasma zinc and IL-6 levels after LPS administration, as well as dysregulated metabolism [25]. Furthermore, the up-regulation of *Slc39a14* was previously reported to be IL-6 dependent [26], suggesting that there may be some level of interdependence between *Slc39a14* and *Il-6* expression. Given this literature, one would anticipate a likely role for *Slc39a14* in host responses to infection. Thus, while functional studies on zinc importers in infectious diseases are limited, SLC39A6, SLC39A8 and/or SLC39A14 represent obvious candidates for regulating zinc trafficking within macrophages, which would likely impact on zinc-regulated signalling, inflammatory responses and microbicidal pathways.

### Copper deficiency compromises immune function and host defence

Copper is required for fundamental metabolic processes, but can be toxic when present in excess [94]. It can exist in two oxidation states in biological systems, cycling between Cu(I) (reduced) and Cu(II) (oxidized) forms, which is harnessed by redox-active enzymes that use copper to accept and donate electrons [95]. Like zinc, copper is required for the development and maintenance of immune function. For example, it is an essential component of the SOD enzyme, which catalyses the production of H_2_O_2_ from superoxide in neutrophils and monocytes [96]. Several lines of evidence indicate that copper deficiency perturbs immune function. Firstly, copper deficiency, in addition to causing neurological dysfunctions, also results in haematological abnormalities, most commonly neutropenia and anaemia [97]. Copper-deficient patients also displayed decreased numbers of myeloid precursors in the bone marrow, as well as vacuolization of these cells [97]. Susceptibility to infections, such as recurrent pulmonary and urinary tract infections and septicaemia has also been reported in Menkes disease [98–101], an X-linked neurodegenerative disease caused by dysregulated copper trafficking [102].

Numerous studies have monitored effects of copper deficiency on immune function in animal models. One of the first reports demonstrated compromised humoral immunity in mice fed on a low copper diet [98]. Subsequent studies have reported increased susceptibility to infectious diseases in copper-deficient animals. For example, mortality rates in response to infection with *Pasteurella haemolytica* were enhanced in mice fed on a copper-deficient diet [103]. Similarly, copper-deficient rats had enhanced mortality rates upon infection with *S.* Typhimurium [104] or *C. albicans* [105], as well as elevated parasitaemia after infection with *Trypanosoma lewisi* [106]. The capacity of leucocytes isolated from copper-deficient livestock to kill *C. albicans* was also significantly reduced [107], which may relate to defects in the functions of neutrophils [108] or macrophages (discussed below).

### Copper as a regulator of macrophage function

**Figure 3:**
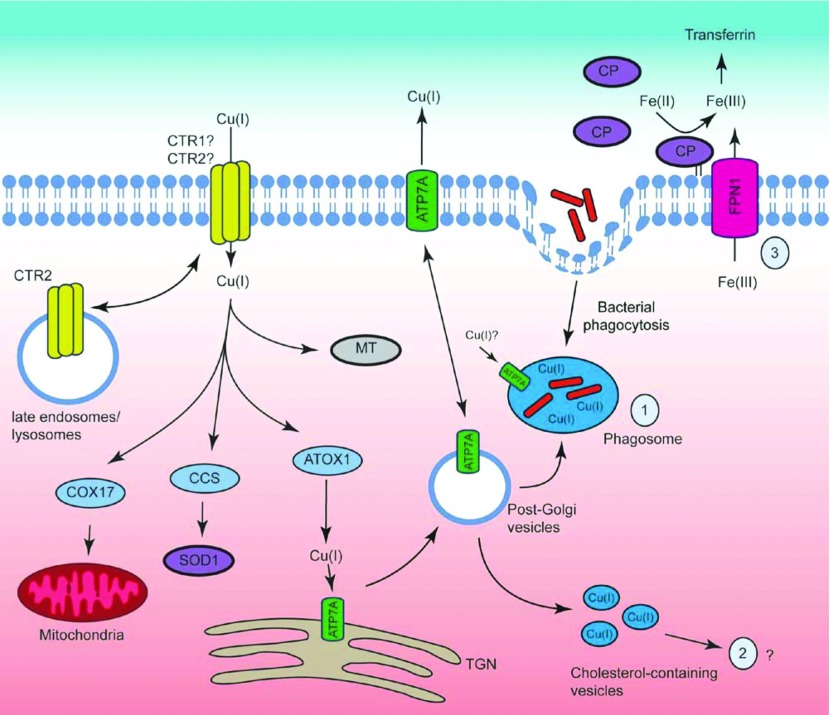
Possible mechanisms by which copper may contribute to macrophage antimicrobial responses Copper is imported into the macrophage by CTR1 and/or CTR2 (most likely CTR1). It is then bound to chaperones (COX17, CCS and ATOX1) or metal-binding proteins such as MT. Copper is transported to the mitochondria for energy production, Cu, Zn-containing SOD1 for cytoprotection or to Atp7a for protein synthesis. IFNγ and/or LPS up-regulate the expression of several copper transport genes in mouse macrophages (e.g. *Ctr1*, *Ctr2*, *Atp7a*) and promote copper uptake into these cells. Possible mechanisms for antimicrobial effects of copper within macrophages include: (1) direct toxicity through Fenton chemistry within phagosomes; (2) vesicular accumulation which may contribute to oxidative stress over a slow time course (e.g. mitochondrial ROS production); and (3) indirect effect via the GPI-anchored form of the copper-containing ferroxidase CP, which promotes FPN1-dependent iron export and thus starves intracellular bacteria of this essential element.

Copper deficiency impacts innate and acquired immune responses, suggesting that copper is likely to regulate the functions of multiple immune cell types [109]. As with the zinc literature, several studies have demonstrated that copper regulates macrophage antimicrobial functions. Macrophages from copper-deficient rats were unimpaired in their ability to phagocytose erythrocytes, but had a defective respiratory burst and were compromised in their ability to kill *C. albicans* [105]. *In vitro* studies also show that copper regulates macrophage antimicrobial pathways. Some of the first evidence for this again came from elemental analysis of macrophage phagosomes; the macrophage activating cytokines IFNγ and TNFα promoted the accumulation of copper within the phagosomes of *M. avium*-infected macrophages [50], suggesting that this metal ion may have some role in macrophage antimicrobial responses. Consistent with this, exogenous copper promoted the bactericidal activity of IFNγ-treated RAW264.7 mouse macrophages against *E. coli*, and this effect was inhibited by the anti-oxidant ebselen [110]. This suggests that copper may contribute to ROS-dependent killing in macrophages, in keeping with the well-known capacity of Cu(I) to catalyse the generation of hydroxyl radical from H_2_O_2_. This study also showed that both IFNγ and LPS regulated copper-trafficking pathways in macrophages. Studies on the intramacrophage pathogen *S.* Typhimurium also provide functional evidence for a role in macrophage antimicrobial pathways. Infection of mouse macrophages with *S.* Typhimurium, as well as treatment with LPS, promoted the accumulation of copper within intracellular vesicles. This response peaked at about 14 h post-stimulation. Furthermore, a cell impermeable copper chelator (bathocuproinedisulfonic acid) reduced vesicular copper accumulation in macrophages, and this impaired the ability of primary mouse macrophages to kill *S.* Typhimurium [111]. The use of a *Salmonella* copper-responsive promoter reporter strain also provided evidence that *S.* Typhimurium within macrophage phagosomes were subjected to an increase in copper levels [112]. Collectively, these studies suggest that copper may regulate both immediate and delayed macrophage antimicrobial pathways. Notably, whereas phagocytosis-induced ROS is an immediate response occurring within the first 30 min of particle uptake, TLR signalling also promotes a slower accumulation of ROS, which is derived from the mitochondria [113]. Hence, it is conceivable that the delayed vesicular accumulation of copper within macrophages in response to LPS could also contribute to this pathway of oxidative stress. It is also possible that inducible copper redistribution contributes to macrophage-mediated host defence by promoting the export of iron (discussed below). The potential mechanisms by which copper could contribute to pathogen clearance by macrophages are outlined in Figure 3.

### What copper transport proteins regulate macrophage functions?

Several of the above studies provide evidence for regulated copper transport in macrophages in response to infectious agents [50,110,111]. Due to the highly toxic nature of Cu(I), copper trafficking must be very tightly regulated. This is achieved through a series of high-affinity copper transport proteins and copper chaperones. Although we have detected moderate increases (<10 fold) in the mRNA expression of the copper chaperones, *Atox1*, *Ccs* and *Cox17* in primary mouse macrophages in response to LPS and *Salmonella* (S. L. Stafford, unpublished work), functional studies of these proteins in macrophage responses to pathogen challenge are lacking. However, emerging evidence implicates some copper transport proteins in macrophage-mediated host defence.

#### CTR (copper transporter) copper importers

The CTR proteins are a family of dedicated high-affinity transport proteins which were first identified in yeast [114], but are conserved through to humans. Mammals have two CTR proteins, CTR1/SLC31A1 and CTR2/SLC31A2 [114], which are thought to form trimeric oligomers in membranes [115]. In cell lines and mouse tissue, CTR1 is found at both the plasma membrane and intracellular vesicles. CTR1 has been shown to traffic from the plasma membrane to endosomal compartments when extracellular copper levels are increased [116,117], which may enable regulatory control of copper uptake. CTR2 shares sequence similarity with CTR1 [114] and is primarily expressed on intracellular vesicles, including late endosomes and lysosomes [118,119]. However, it has also been reported to partially localize to the plasma membrane and facilitate copper uptake [118]. The localization of endogenous CTR1 and CTR2 in unstimulated or activated macrophages has not yet been reported, nor have direct functional studies been performed. Nonetheless, both LPS and IFNγ promoted copper uptake into mouse macrophages [110], and similarly, a cell impermeable copper chelator inhibited the formation of LPS- and *Salmonella*-inducible copper-containing vesicles in macrophages [111]. These stimuli also up-regulated *Ctr1* and *Ctr2* expression in primary mouse macrophages and RAW264.7 cells [110,111], implicating one or both of these transporters in copper uptake by macrophages.

#### ATP7A and ATP7B

ATP7A is a 178 kDa, Cu(I)-transporting P_1B_-type ATPase [120–122], and defects in *ATP7A* are responsible for Menkes disease. ATP7A is expressed in the majority of tissues except for the liver, and has roles in biosynthesis and homoeostasis [123,124]. It is essential for dietary uptake of copper by releasing copper at the basolateral plasma membrane of small intestine cells, delivery of copper to the brain and recovery of copper from proximal tubules of the kidney [125]. Normally, ATP7A is located in the trans-Golgi network, which enables it to supply cuproenzymes [126,127]. When cells are exposed to high copper concentrations, it traffics to the plasma membrane to export the excess copper [128,129]. ATP7A can also deliver copper to intracellular exocytic vesicles and specialised cell compartments such as secretory granules or melanosomes [130]. ATP7B is closely related to ATP7A in both structure and function, and transports copper across membranes in an ATP-dependent fashion [131]. We could not detect *Atp7b* mRNA expression in primary mouse macrophages (S. L. Stafford, unpublished work), but various lines of evidence support a role for *Atp7a* in these cells. Atp7a mRNA and protein expression was robustly up-regulated by LPS and IFNγ in RAW264.7 mouse macrophages [110], and *Salmonella* infection also increased *Atp7a* mRNA levels in primary mouse macrophages [111]. Furthermore, in IFNγ-stimulated RAW264.7 cells, Atp7a partially co-localized to phagosomes during engulfment of latex beads, and knock-down of *Atp7a* impaired the ability of macrophages to kill *E. coli* [110]. Collectively, these data strongly support the idea that macrophages primed by activating agents such as IFNγ can utilize Atp7a for phagosomal delivery of copper to target pathogens for destruction.

### CP (ceruloplasmin)

CP is a multicopper oxidase that is widely distributed in vertebrates. It is mainly produced by the liver and is predominantly found in the plasma [132]. It is a 132 kDa α-2 glycoprotein containing six copper ions, and functions as a ferroxidase [133]. It has roles in iron homoeostasis [134] and antioxidant defence [135], as well as metabolism of copper [136], biogenic amines [137] and NO (nitric oxide) [138]. CP is an acute phase protein [139], and several pro-inflammatory stimuli including IFNγ [140], IL-1, IL-6 [141], TNFα and LPS [142] induce CP synthesis. It is also expressed as a GPI (glycosylphosphatidylinositol)-anchored form [143], and both soluble and GPI-anchored *Cp* mRNA, are robustly induced by LPS in mouse macrophages [111]. More than 95% of serum copper is bound by CP, with the remaining copper being bound primarily with albumin and transcuprein [144]. Surprisingly, aceruloplasminemic patients suffer no copper imbalance; rather, they have impaired iron efflux from cells resulting in complications with iron homoeostasis, which leads to neurodegeneration [145]. CP is thus a key protein that provides a functional link between copper and iron metabolism [146]. It primarily does so through the FPN1 (ferroportin 1) iron exporter; CP ferroxidase activity is required for cell surface FPN1 expression and iron export [147]. Consequently, inducible copper trafficking in macrophages probably enables copper loading into CP, which is required for iron export from macrophages. Iron export from macrophages provides an additional defence mechanism by restricting bacterial growth, as has been clearly demonstrated in the case of *Salmonella* [148]. Interestingly, knock-down of Atp7a in mouse macrophages did not affect CP expression, but reduced CP enzymatic activity [110]. This is consistent with Atp7a contributing to iron export from macrophages, in addition to direct delivery of copper to the phagosome.

### Interplay between zinc and copper in macrophage-mediated host defence

As reviewed above, there are remarkable parallels between zinc and copper with respect to macrophage antimicrobial pathways. Both these metal ions promote macrophage antimicrobial responses, and their intracellular localizations are dynamically regulated in macrophages responding to pathogen challenge. Furthermore, both zinc and copper can be delivered to the macrophage phagosome, raising the question of whether there is interplay between the two ions in macrophage-mediated host defence. Both zinc and copper are soft metal ions that can inactivate the exposed Fe-S cluster of key bacterial dehydratase enzymes, including 6-phosphogluconate dehydratase (Entner–Douderoff Pathway), fumarase A [TCA (tricarboxylic acid) cycle] and isopropylmalate isomerase (leucine biosynthesis) [149]. This property may be harnessed within the intramacrophage environment, perhaps in a synergistic fashion. The pro-oxidant properties of copper ions have already been noted, while zinc, although not a redox active metal ion, has the ability to bind to protein thiol groups in the cell. It is established that zinc inhibits thioredoxin reductase, for example [150], and this could perturb antioxidant defences linked to thioredoxins. Thus, copper and zinc could independently enhance oxidative stress during macrophage antimicrobial responses, even though zinc is more generally associated with antioxidant effects. The importance of intracellular copper and zinc buffering by low molecular mass thiols in the protection against metal-ion toxicity was recently demonstrated for pneumococcus, where it was shown that mutants lacking the ability to import glutathione were hypersensitive to copper and zinc [151].

## CONCLUSIONS AND FUTURE DIRECTIONS

The interplay between zinc and macrophages is complex. Zinc appears to enhance the microbicidal activity of macrophages, while at the same time limiting excessive inflammatory responses that may be deleterious to the host. The redistribution of the intracellular zinc pool to phagosomes and other vesicular compartments may enable macrophages to harness the activity of this metal ion for microbial destruction. Conversely, this sequestration away from the cytoplasm, as well as inducible export of cytoplasmic zinc from macrophages, may also enable these cells to starve certain pathogens of zinc to limit growth. The specific transporters involved in trafficking zinc within and out of macrophages are not well understood, but there are several obvious candidates within the SLC30A (e.g. SLC30A1, SLC30A4, SLC30A6) and SLC39A (e.g. SLC39A6, SLC39A8, SLC39A14) families. Functional analysis of these genes should reveal new insights into macrophage responses to pathogen challenge. Macrophages may utilize copper in host defence strategies through several mechanisms including acute and delayed generation of ROS, as well as iron export as a means of limiting bacterial growth. Further experimental evidence is still required to support each of these potential mechanisms. Many of the copper transport genes have also been implicated in macrophage-mediated host defence (e.g. CTR1, CTR2, ATP7A, CP). Analysis of macrophage-specific knock outs for the above zinc and copper transport genes is now required to determine their *in vivo* roles in macrophage antimicrobial pathways. An understanding of zinc and copper trafficking in macrophages in response to distinct classes of pathogens (e.g. Gram-positive against Gram-negative bacteria, cytoplasmic against vesicular intramacrophage pathogens) may also yield insights into the requirements for these pathways in different infectious disease settings.
